# National

**Published:** 1896-04

**Authors:** 


### NATIONAL.

### CIVIL-SERVICE EXAMINATION FOR MEAT-INSPECTOR.

WRITE a letter, of not less than one hundred and twenty-five words, addressed to the United States Civil Service Commission, Washington, D. C., and briefly state your views on the general influence of the cold-storage system, on the quality, preservation, and handling of meats. Describe the alimentary canal of a cow. Define myocarditis, exostosis, ascites, and septicæmia. Give the cellular elements of the blood and their functions. In what disease is the spleen affected? Give example. What diseases cause skin-eruption? What lesions simulate tuberculosis? Name the causes of hæmoglobinuria other than infectious. Describe the condition of a slaughtered carcass of a healthy animal; of one suffering from an infectious disease; one that died a natural death. What are the products of an inflammation of a serous membrane? Under the present law what disposition is made of diseased meat?

### RANK IN THE ARMY.

CHAIRMAN TURNER has found active and strong support in Virginia through the efforts of one of Virginia's veterinarians. Representative Tyler, of that State, a member of the House Military Committee, favors the passage of this measure.

Every Pennsylvania Representative in the House at Washington has been addressed in the interest of the Army Veterinary Bill by joint letters from the Presidents of the United States, Pennsylvania State and Keystone Veterinary Medical Associations, through Messrs. Hoskins, Pearson, and Hart, representing the latter Association.

Secretary Merillat, of the McKillip Veterinary College, has become an interested worker in favor of army legislation that shall place the veterinarian in his proper sphere.

The War Department has returned the Army Veterinary Bill to the House Military Committee with its approval. Adjutant-General Ruggles placed his favorable approval to the Senate Bill when received for his opinion. General Miles, on receiving the proposed Army Legislation Bill, drew up a new one, allowing one veterinary surgeon to each six troops of cavalry, and one to each six batteries of horse or light artillery. They were to be staff officers and rank as second lieutenants, to receive $1200 per year salary, and 10 per cent. additional for each five years of service, examinations to precede each increase. This bill gave rank, privileges, allowances on retirement, pension, etc. Those in the service for fifteen years or over to be reappointed without examination. Those less than this period to pass an examination such as the Secretary of War may direct. In case of failure to pass said examination to be given three months' leave of absence and be discharged. The last clause required all horses bought or sold to be inspected by the army veterinarians. Both bills on reaching the Quartermaster-General's office met his disapproval, and he recommended the discharge of all present veterinary surgeons in the service and the employing of ten civilians by contract. The Assistant Secretary of War then took up the several recommendations and approved the Quartermaster-General's report in the face of the favorable approval of Generals Miles and Ruggles. It will be recalled that at present the purchase and sale of all horses goes through the Quartermaster-

General's office, and he may or may not request the service and opinion of the veterinarian at will.

